# Thermoelectric Performance Enhancement in Off-Stoichiometric Ag–Se Alloys via Composition Imbalance and Vacancy Suppression

**DOI:** 10.1021/acsomega.5c12125

**Published:** 2026-09-14

**Authors:** Dieter Rahmadiawan, Wen-Hui Lin, Jen-Fin Lin, Shih-Chen Shi, Tao-Hsing Chen, Hao-Tung Lin

**Affiliations:** † Department of Mechanical Engineering, 34912 National Cheng Kung University, Tainan 70101, Taiwan; ‡ Department of Mechanical Engineering, Universitas Negeri Padang, Padang, Sumatera Barat 25173, Indonesia; § Department of Mechanical Engineering, 517768 National Kaohsiung University of Science and Technology, Kaohsiung 80778, Taiwan; ∥ 63129 Industrial Technology Research Institute, Green Energy & Environment Research Laboratories, Hsinchu 31040, Taiwan

## Abstract

This study investigates the thermoelectric performance of off-stoichiometric Ag–Se alloys through composition-imbalance engineering. By varying the Ag-to-Se ratio, we successfully tuned the carrier concentration and mobility by suppressing Se-vacancy formation and reducing electron scattering. Simultaneously, grain refinement induced by excess Se enhanced phonon scattering, thereby lowering the thermal conductivity. Structural and phase analyses confirmed that Ag_2_Se remained the dominant phase across compositions. These findings highlight composition imbalance as a simple yet effective strategy for tuning thermoelectric performance in Ag–Se systems. This work offers a new pathway for designing high-performance, low-temperature thermoelectric materials.

## Graphical abstract

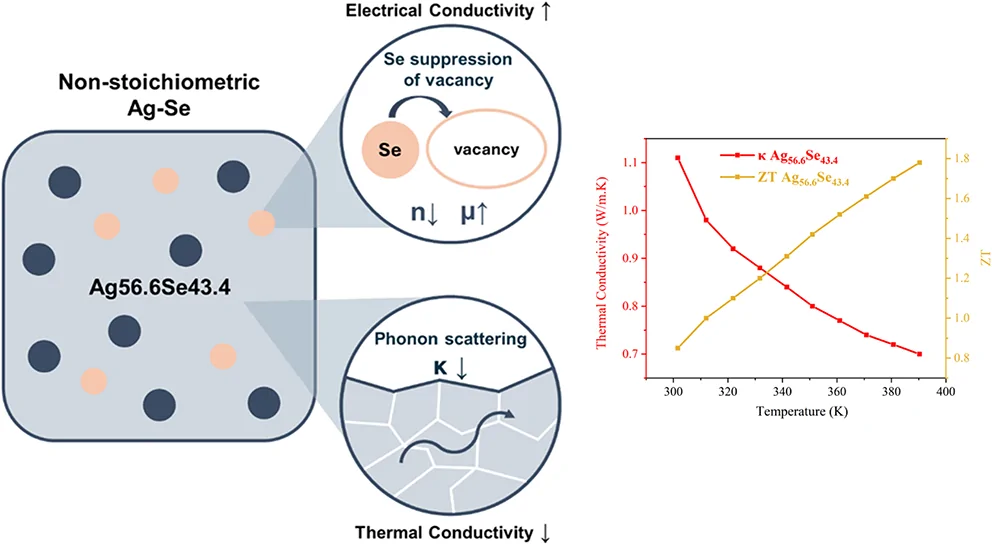

## 1 Introduction

The growing energy demand of the 21st century, driven by rapid industrialization and digital reliance, has exposed the limitations of conventional, nonrenewable energy sources. Their environmental impact and economic instability highlight the urgent need for clean, sustainable alternatives.^1–5^ Metallic materials have long played a central role in engineering and industry due to their excellent fracture resistance, thermal stability, and corrosion resistance. Traditional alloys such as steels and aluminum alloys typically consist of one major element alloyed with one or two minor elements to enhance specific properties.^6^ However, the addition of multiple alloying elements often leads to the formation of complex microstructures or intermetallic compounds, which may degrade mechanical performance and induce embrittlement. These limitations have prompted the search for novel materials and design strategies that not only retain structural integrity but also contribute to functional performance in energy-related applications.

To address the pressing need for energy efficiency and sustainability, advanced materials capable of converting waste heat into usable electricity are gaining attention. Among them, thermoelectric materials have emerged as promising candidates, offering unique advantages for applications in cooling, sensing, energy harvesting, and solid-state power generation systems.^7,8^ Their ability to directly convert waste heat into electricity makes them promising candidates for enhancing energy efficiency and enabling sustainable energy solutions in automotive systems, wearable electronics, and solar energy platforms.^9,10^ The global thermoelectric materials market is projected to reach USD 74 billion by 2024 and USD 155.49 billion by 2033, growing at a compound annual rate of 8.6%.^11^

The efficiency of thermoelectric materials is evaluated by the dimensionless figure of merit (ZT), defined as ZT = S^2^σT/κ, where S is the Seebeck coefficient, σ is the electrical conductivity, T is the absolute temperature, and κ is the total thermal conductivity.^12,13^ A high ZT requires the simultaneous achievement of a high S, high σ, and low κ.^14,15^ However, these parameters are interdependent, making it difficult to optimize them concurrently.

Among the various thermoelectric materials, traditional systems such as Bi_2_Te_3_-, PbTe-, and SnSe-based compounds have been extensively studied due to their high thermoelectric performance. However, these materials often suffer from limitations including toxicity, high cost, scarcity of elements, and poor stability under operating conditions.^16^ These drawbacks have driven the search for alternative thermoelectric materials that are environmentally benign, cost-effective, and suitable for low-temperature applications.

Ag_2_Se demonstrates excellent potential among low-temperature thermoelectric materials due to its narrow bandgap, complex band structure, high σ, moderate S, and exceptionally low κ.^17,18^ Its superior transport properties are attributed to the disordered motion of Ag^+^ ions and Se vacancies. At approximately 407 K, Ag_2_Se undergoes a phase transition from an orthorhombic α-phase to a superionic cubic β-phase, where Ag^+^ ions exhibit high mobility within the Se lattice. Moreover, Ag and Se are more abundant and less toxic than conventional thermoelectric elements like Te and Bi, making Ag_2_Se a more sustainable alternative.^19,20^ Nonetheless, its reported ZT value remains limited to about 1.0,^21^ below practical application thresholds.

Several studies have investigated the effect of Ag-to-Se stoichiometry on thermoelectric performance. Guo et al.^22^ demonstrated that slight Se excess in Ag_2_Se_1+x_ stabilized the pure phase and improved ZT to ∼0.72 in the 310–380 K range. Gáborová et al.^23^ used mechanochemical synthesis and showed that Se enrichment significantly impacted carrier concentration and thermoelectric behavior. Wang et al.^24^ reported that excess Se can influence both carrier concentration and mobility, depending on the compositional regime and defect structure, collectively affecting electrical performance.

This study hypothesizes that excess Se in Ag_2_Se suppresses the formation of Se vacancies, leading to a reduction in free electron concentration while enhancing carrier mobility by minimizing vacancy-induced scattering. The decrease in carrier concentration is expected to increase the absolute Seebeck coefficient due to its inverse relationship with thermopower, whereas improved mobility helps maintain electrical conductivity despite reduced carrier concentration. Consequently, an optimized balance between Seebeck coefficient and electrical conductivity is anticipated, resulting in an enhanced power factor.

In addition, Se surplus is expected to promote grain refinement, which increases phonon scattering and effectively reduces lattice thermal conductivity. By simultaneously improving carrier transport and suppressing lattice thermal conductivity through a non-stoichiometric composition strategy, the overall thermoelectric performance, as reflected by ZT, is expected to improve.

Compared with previous studies, which generally reported an increase in carrier concentration with moderate Se enrichment, the present work reveals an opposite trend in a more Se-rich compositional regime, where carrier concentration decreases while mobility is enhanced. This difference highlights the existence of distinct defect and transport regimes in Ag–Se systems.

The novelty of this study lies in demonstrating that compositional imbalance not only tunes carrier concentration but also enables simultaneous optimization of carrier mobility and Seebeck coefficient, resulting in a significantly enhanced power factor. This provides new insight into the role of Se enrichment beyond the conventional understanding reported in earlier works.

## 2 Materials and Methods

### 2.1 Materials

Commercially available materials were used to prepare the Ag–Se samples. Silver powder (100 mesh, 99.95%; Merck, Germany) and selenium powder (100 mesh, 99.5%; Merck, Germany) served as the primary precursors. Additional materials included stainless steel balls (3 mm diameter), 80 mL jars, 100–500 mesh sieves, boron nitride spray (COMBAT, France), carbon paper, and 20 mm graphite dies. These components supported the synthesis and processing of off-stoichiometric Ag–Se alloys.

### 2.2 Mechanical Alloying

Ag and Se powders were mixed by weight according to Table 1. The mixtures were loaded into milling jars along with 100 g of steel balls and processed at 450 rpm for 10 min using a planetary ball mill (PULVERISETTE 6, Fritsch GmbH, Germany) to produce alloyed powders. This step ensured homogeneous mixing and initiated solid-state reactions.

**Table 1:** Sample Codes and Sintering Conditions of Off-Stoichiometric Ag–Se Alloys

Sample code	Ag: Se (wt % : wt %)	Sintering temperature (K)	Pressure (MPa)	Heating rate (°C/min)	Holding time (min)
Ag_50_Se_50_	50 : 50	573	55	50	10
Ag_56.6_Se_43.4_	56.6 : 43.4				
Ag_60_Se_40_	60 : 40				
Ag63.3Se36.7	63.3 : 36.7				
Ag_70_Se_30_	70 : 30				

### 2.3 Spark Plasma Sintering (SPS)

The milled powders were sieved to a particle size of ≤25 μm using 100–500 mesh sieves. Four pieces of carbon paper were cut to fit a 20 mm graphite mold. Boron nitride spray was applied to the inner mold surfaces and allowed to dry to prevent sticking. The mold was assembled with alternating layers of carbon paper and powder, then pre-pressed at 100 kgf for 2 min per side. The assembly was placed into the SPS chamber (SPS-515S, Dr. Sinter*LAB, Japan) and sintered at 573 K under a uniaxial pressure of 55 MPa, with a heating rate of 50 °C/min for 10 min. Sintering conditions are summarized in Table 1. After natural cooling, the samples were demolded, cleaned, and polished for further characterization.

### 2.4 Characterization

Sample properties were characterized using multiple analytical techniques. Phase composition was examined by X-ray diffraction (XRD, D2 PHASER, Bruker, Germany), and the diffraction patterns were analyzed using standard reference data from the International Centre for Diffraction Data (ICDD) database. Surface morphology and elemental distribution were analyzed using scanning electron microscopy (SEM, SU-5000, Hitachi) equipped with energy-dispersive spectroscopy (EDS).

Surface morphology and elemental distribution were analyzed using a field-emission scanning electron microscope (FE-SEM, SU-5000, Hitachi, Japan) equipped with energy-dispersive spectroscopy (EDS) for microstructural observation and elemental analysis. High-resolution structural analysis was further performed using an ultrahigh-resolution transmission electron microscope (TEM) (JEM-2100F CS STEM, JEOL, Japan). Selected area electron diffraction (SAED) was conducted to analyze the crystallographic structure and phase identification.

For thermoelectric measurements, the sintered samples were cut into rectangular bar-shaped specimens with dimensions ranging from 1 × 1 × 8 mm^3^ to 4 × 4 × 15 mm^3^, in accordance with the requirements of the ZEM-3 system (ULVAC, Japan). Electrical conductivity and Seebeck coefficient were measured in the temperature range of 300–390 K using the ZEM-3 system, with a thermocouple spacing of 8 mm.

Prior to measurement, the sample surfaces were polished to ensure good electrical and thermal contact. Measurements were conducted under a low-pressure helium atmosphere to minimize oxidation. No visible oxidation, surface degradation, or color change was observed after measurement, indicating good thermal stability within the tested temperature range.

Thermal conductivity was measured using a physical property measurement system (PPMS-16T, Quantum Design, USA) equipped with a thermal transport option, using samples with dimensions of 3 × 3 × 5 mm^3^. In this method, thermal conductivity was determined directly from the applied heat flow and the resulting temperature gradient according to Fourier’s law. Therefore, thermal diffusivity was not separately measured, and thermal conductivity was not calculated using the relationship κ = αρC_p_.

The relative density of the stoichiometric Ag_2_Se sample was determined by the Archimedes method based on buoyancy measurements in deionized water, yielding a relative density of 98.2%. Hall measurements (HALL8800, SWIN) were conducted at room temperature to determine carrier concentration and mobility, using samples with dimensions ranging from 5 × 5 × 2 mm^3^ to 20 × 20 × 2 mm^3^. The ZT value was calculated using the measured Seebeck coefficient, electrical conductivity, and thermal conductivity according to ZT = S^2^σT/κ equation.

The measurement uncertainty of the ZEM-3 system, particularly for the derived power factor, is approximately ±7%, as reported in the literature.^25^ All measurements were performed once for each sample under controlled conditions. The obtained values were verified to be physically reasonable and consistent with expected trends and literature reports, indicating reliable measurement outcomes within the instrument uncertainty range.^26–28^

## 3 Results and Discussion

### 3.1 Off-Stoichiometric Ag–Se Alloys

#### 3.1.1 Phase and Microstructure Analysis of Off-Stoichiometric Ag–Se Alloy Powders

XRD analysis investigated the phases in ball-milled powders and SPS-sintered off-stoichiometric Ag–Se alloys. As shown in Figure 1, all mechanically alloyed powders exhibited the Ag_2_Se phase (ICDD PDF No. 24–1041), indicating that the ball milling process successfully promoted Ag_2_Se formation.^29^ As the Ag–Se ratio deviated from the stoichiometric value (73 wt %: 27 wt %), the XRD signal became increasingly noisy, suggesting incomplete reactions in Se-rich compositions. These results indicate that the Ag_2_Se phase formed consistently across all compositions; however, crystallinity was reduced in samples with excess Se, likely due to incomplete phase formation or amorphous byproducts.

**Figure 1:**
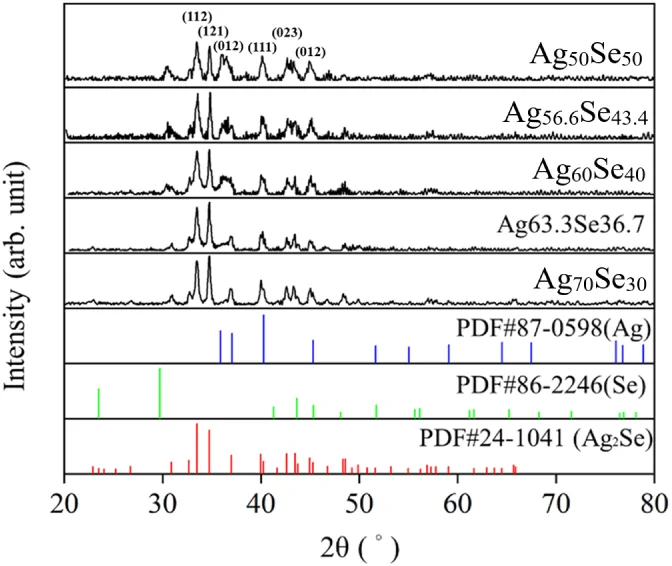
XRD patterns of ball-milled off-stoichiometric Ag–Se powders.

XRD patterns were also collected from the top, bottom, and cross-sectional areas of the sintered samples. The reference diffraction patterns shown at the bottom of Figure 1 correspond to orthorhombic Ag_2_Se (red, ICDD PDF No. 24-1041), elemental Se (green, ICDD PDF No. 86-2246), and metallic Ag (blue, ICDD PDF No. 87-0598). These reference patterns were included to facilitate phase identification and to distinguish the Ag_2_Se matrix from possible secondary phases or residual elemental constituents in the off-stoichiometric Ag–Se alloys. As shown in Figure 2, all compositions exhibited the orthorhombic Ag_2_Se phase with space group P2_1_2_1_2_1_ (ICDD PDF No. 24–1041), with dominant diffraction peaks observed at 2θ = 33.5° and 34.7°, corresponding to the (112) and (121) planes.

**Figure 2:**
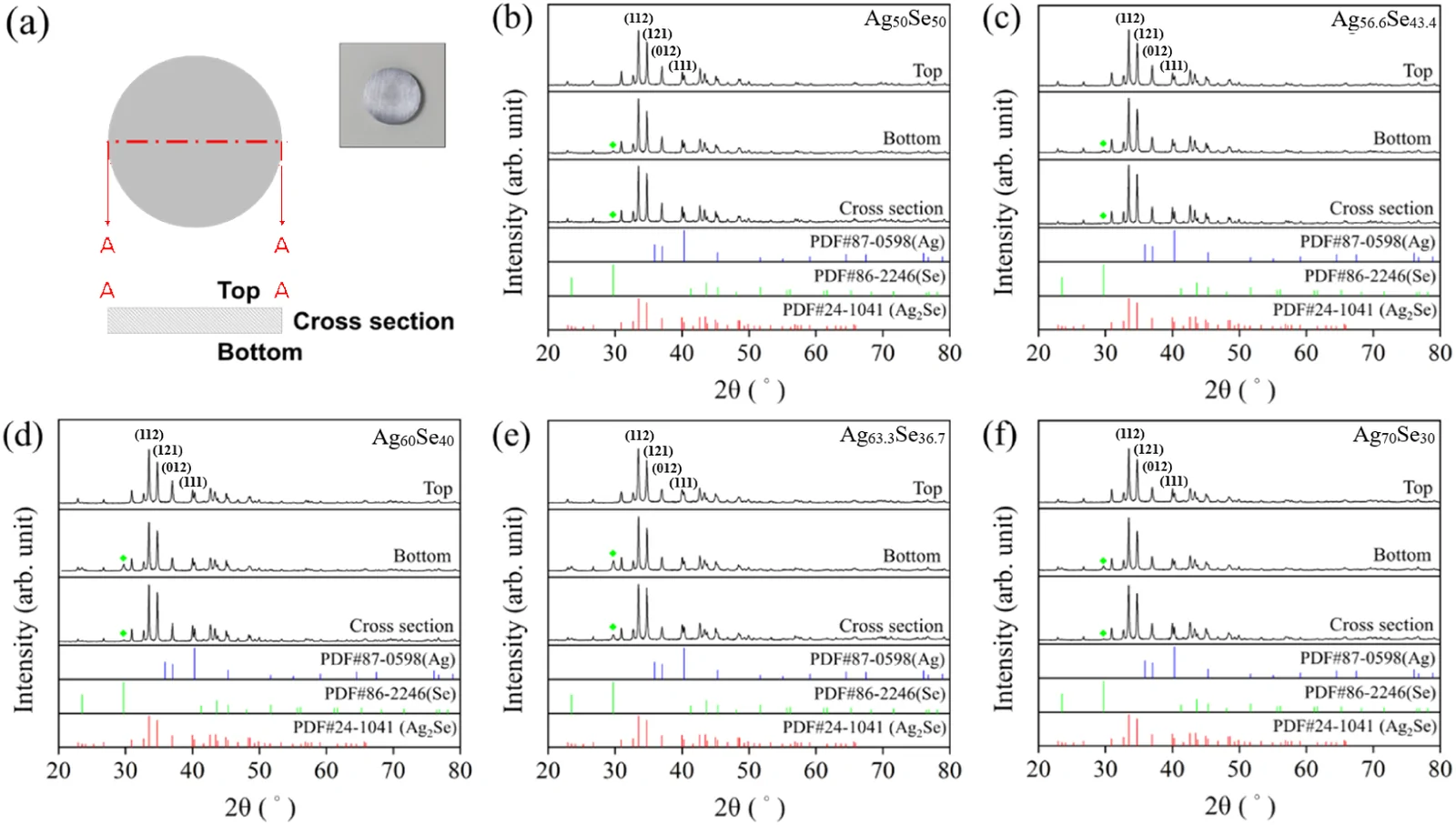
(a) Schematic and photo of sample cross-section, (b)-(f) XRD patterns from various regions of sintered Ag–Se alloys.

As shown in Table 2, the intensity of Ag_2_Se diffraction peaks decreased as the Ag-to-Se ratio deviated from the stoichiometric composition. This result confirms that excess Se hinders complete formation of the Ag_2_Se phase, leading to reduced crystallinity and structural non-uniformity within the sintered pellets.

**Table 2:** Crystallite Size and XRD Peak Intensity of Sintered Ag–Se Samples

	Crystallite size (nm)		Intensity	
Sample code	(112)	(121)	(112)	(121)
**Ag**_**50**_ **Se**_**50**_	39	38	738	500
**Ag**_**56.6**_ **Se**_**43.4**_	35	33	743	687
**Ag**_**60**_ **Se**_**40**_	39	41	1003	846
**Ag**_**63.3**_ **Se**_**36.7**_	53	53	1015	1052
**Ag**_**70**_ **Se**_**30**_	66	80	1245	1054

Elemental and microstructure analyses were conducted to support the XRD findings. Figure 3 shows the EDS elemental maps for the sample cross sections. Se precipitation was observed in Se-rich samples, particularly concentrated in the lower part of the specimens. EDS results confirmed a higher Se weight percentage at the bottom region. These findings align with the XRD results in Figure 2, where excess Se appeared in non-stoichiometric samples. This suggests that Se segregation occurs as a consequence of excess Se content, adversely affecting the structural and compositional uniformity of the sintered alloys.

**Figure 3:**
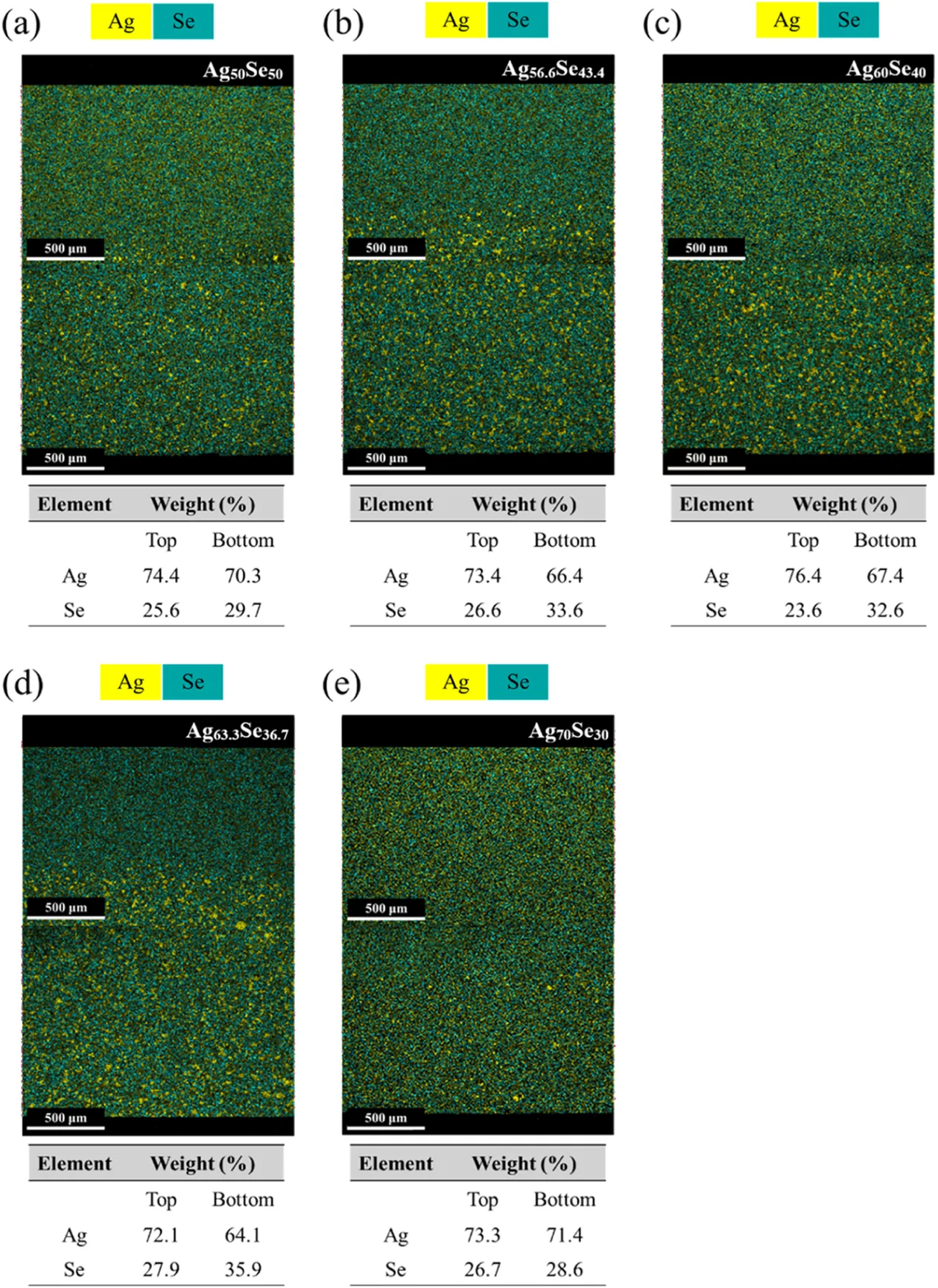
Cross-sectional EDS elemental maps of sintered Ag–Se samples with varying compositions: (a) Ag_50_Se_50_, (b) Ag_56.6_Se_43.4_, (c) Ag_60_ Se_40_, (d) Ag_63.3_Se_36.7_, and (e) Ag_70_Se_30_.

TEM and SAED analyses were conducted to further verify the internal structure and crystallinity of the samples. Figure 4 shows selected-area diffraction patterns and high-resolution images for Ag_56.6_Se_43.4_ and Ag_63.3_Se_36.7_ samples. SAED patterns in Figure 4(a) and 4(c) displayed uniform orthorhombic Ag_2_Se diffraction rings along [111] and [001] zone axes. HR-TEM images in Figure 4(b) and 4(d) revealed well-defined lattice fringes indicative of good crystallinity. These results confirm that Ag_2_Se forms with a high degree of structural order, even in compositions with deviations from stoichiometry.

**Figure 4:**
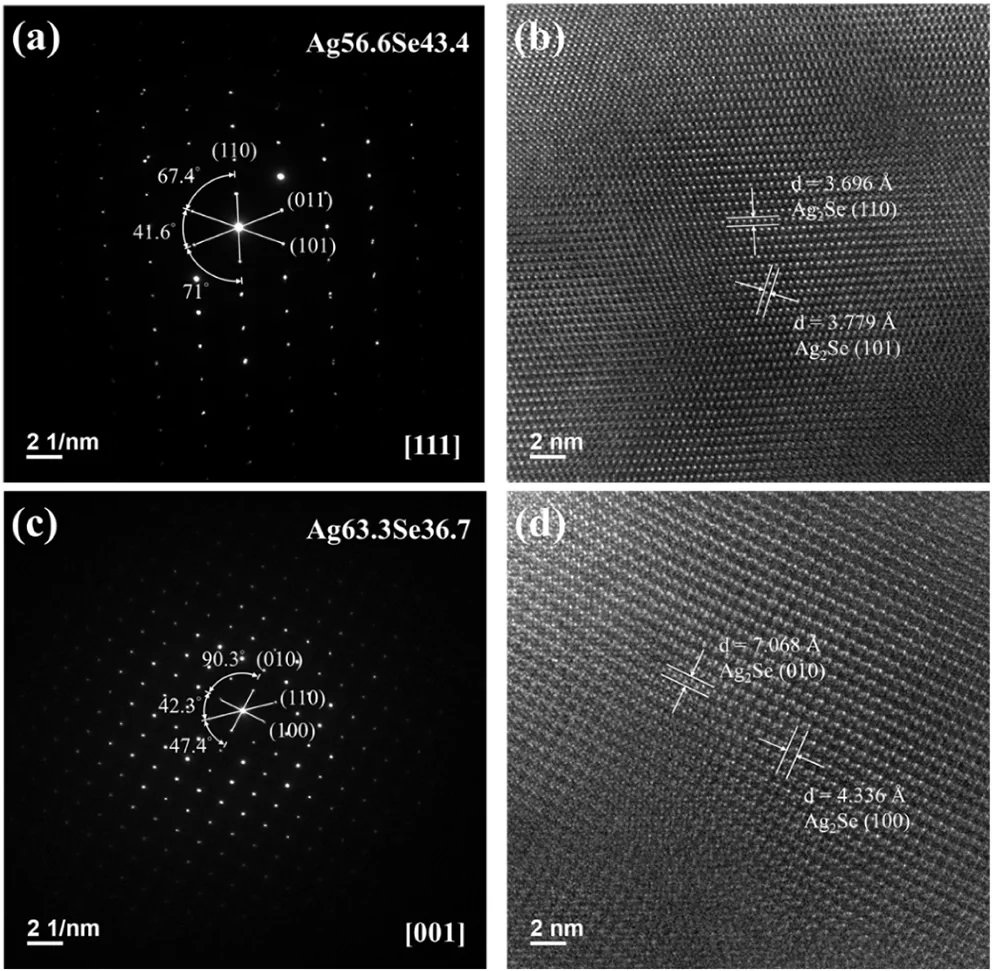
(a), (c) SAED and (b), (d) HR-TEM images of sintered Ag_56.6_Se_43.4_ and Ag_63.3_Se_36.7_ samples.

SEM analysis revealed elevated Se content at the bottom of the samples. This behavior is explained by the physical properties of Se, which has a melting point of 221 °C.^30^ During sintering, unreacted Se partially melted and gradually infiltrated the bulk under gravity, as shown in Figure 5. The molten Se solidified upon cooling, resulting in higher Se content in the lower region. This result indicates that Se’s physical behavior during sintering leads to compositional stratification in the sample.

**Figure 5:**
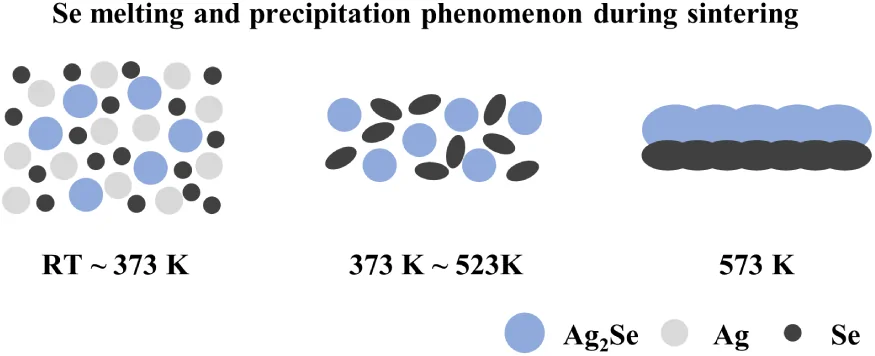
Mechanism of Se melting and redistribution during sintering.

In addition, Se vacancies formed due to the volatility of Se during high-temperature sintering. When excess Se is present, it likely replenishes these vacancies during sintering and cooling. This mechanism relates to the intrinsic crystal structure of Ag_2_Se and its vacancy behavior,^31,32^ as illustrated in Figure 6. No additional diffraction features or superlattice reflections were observed in the SAED and HRTEM results (Figure 4), indicating the absence of long-range defect ordering or secondary phases.

**Figure 6:**
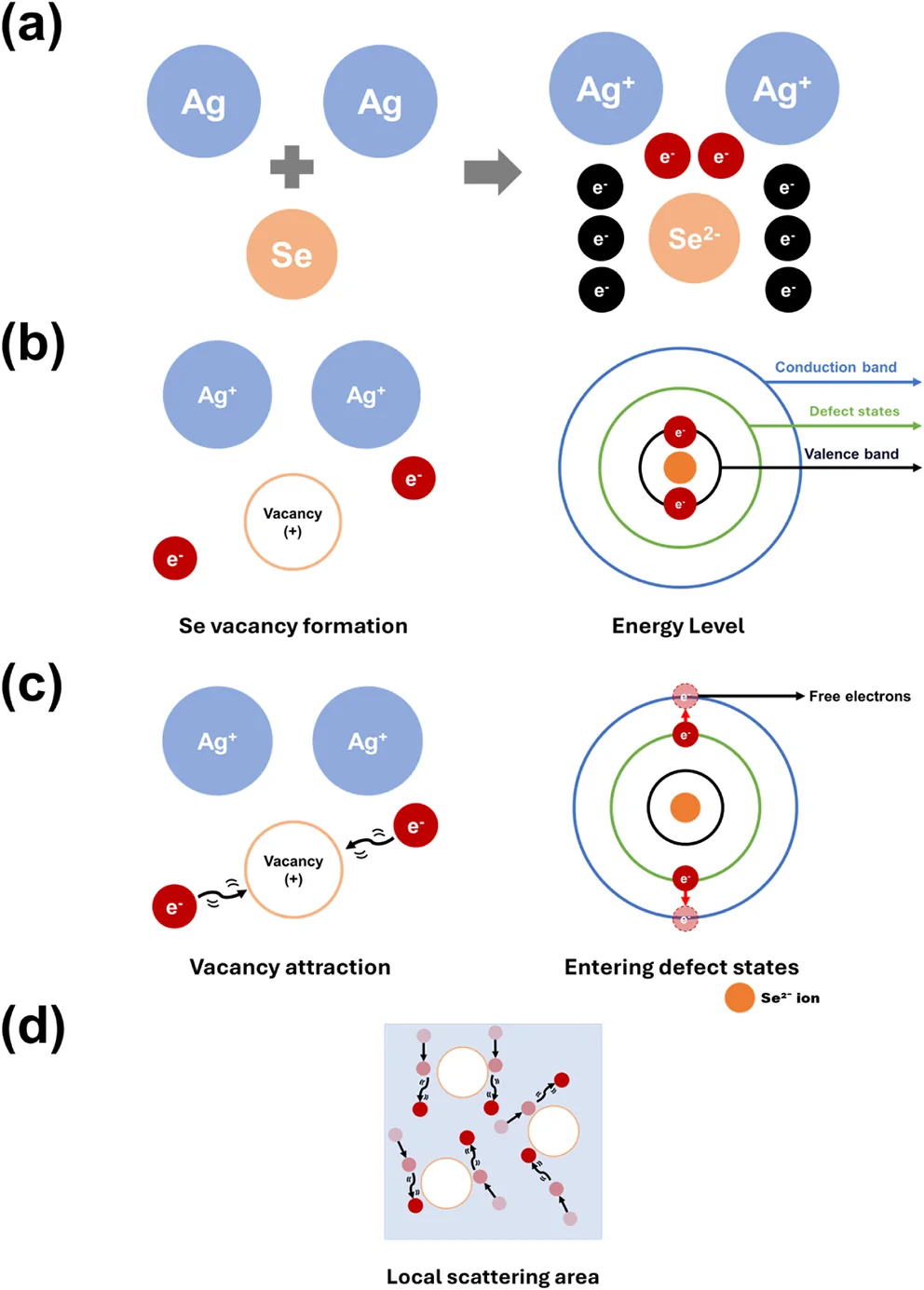
(a) Ionic bonding in Ag_2_Se, (b) Se vacancy energy levels, (c) electron capture at vacancy sites, and (d) vacancy-induced electron scattering.

Figure 6(a) schematically illustrates the ionic bonding between Ag and Se atoms. Ag has an atomic number of 47 and configuration [Kr] 4d^10^5s^1^, contributing one valence electron. Se has atomic number 34 and configuration [Ar] 3d^10^4s^2^4p^4^, requiring two electrons to complete its octet.

Each Ag atom donates one electron to become Ag^+^ ([Kr] 4d^10^), and Se gains two electrons to form Se^2–^ ([Ar] 3d^10^4s^2^4p^6^). Therefore, Ag_2_Se forms stable ionic bonds, as expressed in eq 1:

1 $${\mathrm{Ag}}_{2}\mathrm{Se}=2{\mathrm{Ag}}^{+}+{\mathrm{Se}}^{2‐}$$

Some Se atoms volatilized during sintering, leading to the formation of Se vacancies.^33,34^ Figure 6(b) shows that a missing Se^2–^ leaves behind a positively charged vacancy, attracting nearby electrons. These electrons are easily trapped in the defect levels (Figure 6(c)), which act as intermediate states that lower the transition energy from valence to conduction bands. This result facilitates free electron generation.^35^ However, if vacancy concentration is excessive, localized scattering centers form and impede electron transport, as depicted in Figure 6(d).^36^ Thus, moderate vacancy formation may enhance conductivity, but excessive vacancies degrade carrier mobility.

Assuming each Se vacancy releases two free electrons, the relationship between carrier concentration (n) and vacancy concentration (N_V_) is given by eq 2:

2 $$N_{V}\approx \frac{n}{2}$$

where n is the carrier concentration obtained from Hall measurements (cm^–3^), the derived N_V_ shares the same unit, enabling direct comparison.

#### 3.1.2 Thermoelectric Properties of Off-Stoichiometric Ag–Se Alloys

Carrier concentration and mobility were measured at room temperature for all sintered samples. Figure 7 shows that when the Ag–Se ratio approached the stoichiometric composition of Ag_2_Se, the carrier concentration increased while mobility decreased. This behavior is attributed to the intrinsic structure and vacancy mechanism of Ag_2_Se. As the Ag–Se ratio deviated from stoichiometry, excess Se tended to precipitate and fill existing Se vacancies, thereby reducing free electron generation and lowering the carrier concentration.

**Figure 7:**
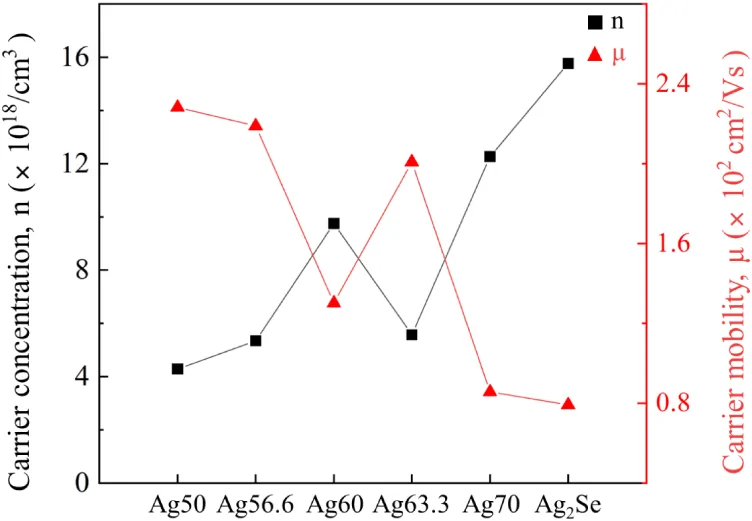
Carrier concentration and mobility of sintered Ag–Se alloys.

It should be noted that the effect of excess Se on carrier concentration is not universal and may vary depending on the compositional range and processing conditions, which may explain differences compared to previous reports.^24^ Simultaneously, the reduction in vacancy-induced scattering allowed carrier mobility to increase. Figure 8(a) further confirms this inverse relationship between carrier concentration and mobility.

**Figure 8:**
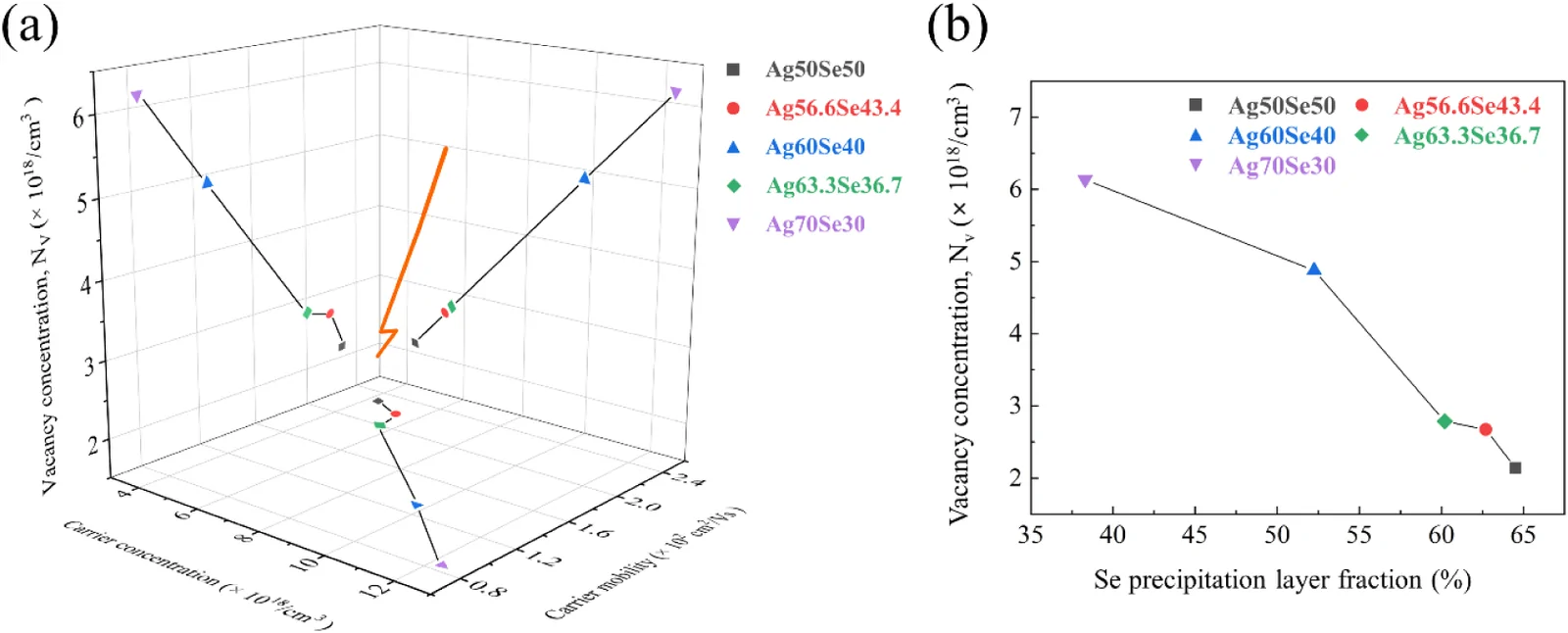
(a) 3D plot of vacancy concentration vs. carrier properties, (b) Se-rich layer thickness vs. vacancy concentration.

However, the transport properties do not follow a strictly monotonic trend with composition. In particular, the Ag_63.3_Se_36.7_ sample exhibits a deviation from the general trend. This behavior is likely associated with the complex interplay between defect chemistry, local compositional heterogeneity, and microstructural evolution. Possible non-uniform distribution of excess Se or localized phase segregation may result in a higher apparent Se-precipitate fraction compared to Ag_60_Se_40_, despite the lower nominal Se content. Such local variations can significantly influence carrier scattering and transport properties, leading to non-monotonic behavior.

Additionally, Figure 8(b) reveals thicker Se-rich layers in the Ag_50_Se_50_, Ag_56.6_Se_43.4_, and Ag_63.3_Se_36.7_ samples, indicating that a greater number of vacancies were filled, which in turn suppressed carrier generation and enhanced mobility.

Thermoelectric parameters were evaluated in the temperature range of 300–390 K. Figure 9(a) shows that electrical conductivity (σ) increased with temperature, exhibiting a typical semiconducting behavior resulting from the thermal excitation of charge carriers.^37,38^ The Ag_56.6_Se_43.4_, Ag_60_Se_40_, and Ag_63.3_Se_36.7_ exhibited higher conductivity (∼13 × 10^4^ S/m), while Ag_50_Se_50_ and Ag_70_Se_30_ were lower (∼10 × 10^4^ S/m). As defined in eq 3, electrical conductivity depends on the carrier concentration and mobility:^39^

3 σ=neμ

where n is the carrier concentration (cm^–3^), e is the elementary charge (1.6 × 10^–19^ C), and μ is the carrier mobility (cm^2^/V·s).

**Figure 9:**
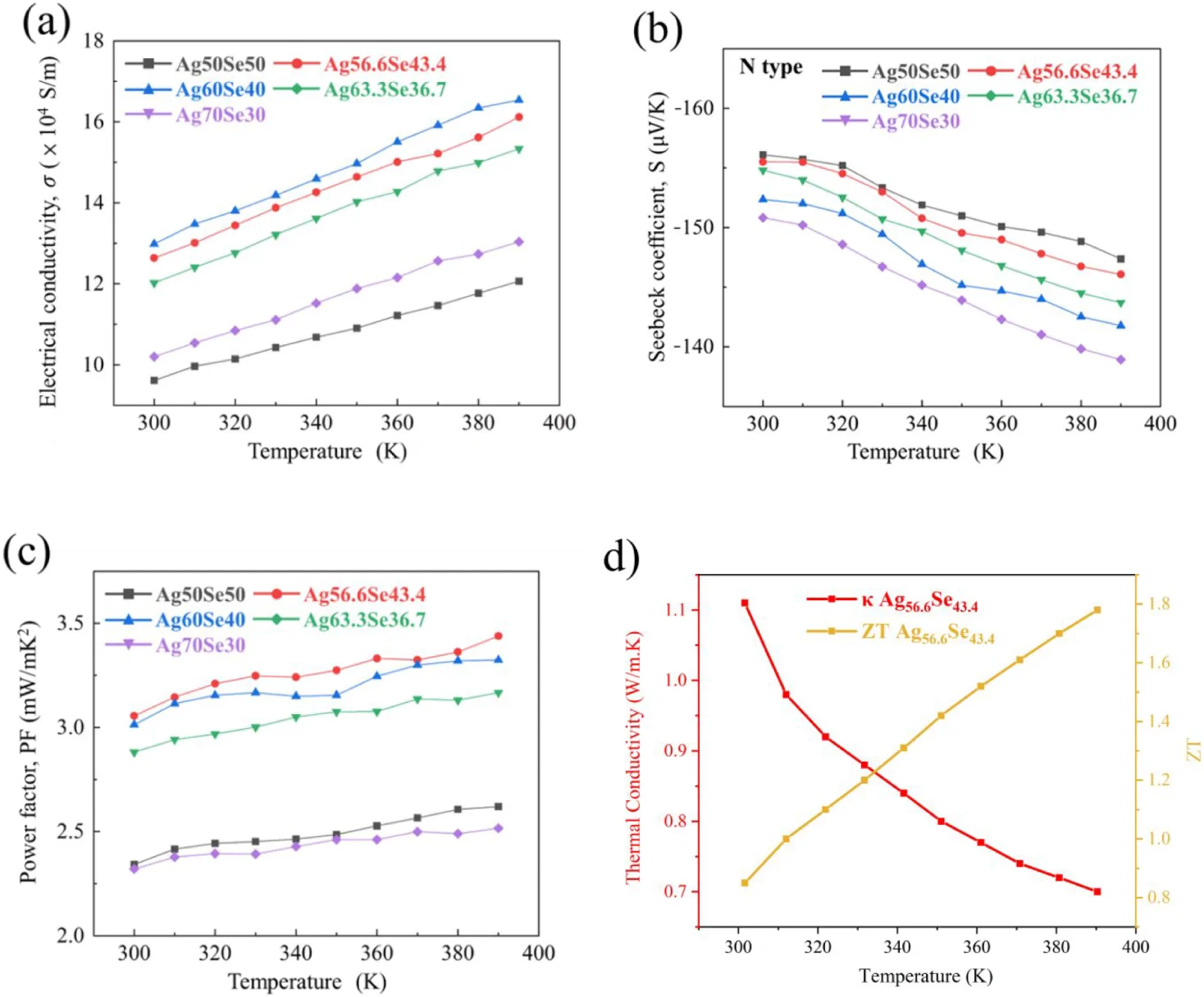
(a) Electrical conductivity vs. temperature, (b) Seebeck coefficient vs temperature, (c) Power factor vs temperature, and (d) thermal conductivity and ZT value of Ag_56.6_Se_43.4_.

Se precipitation decreased vacancy concentration, which reduced n but increased μ. In intermediate compositions, this balance yielded higher conductivity.

Figure 9(b) shows that all samples exhibited negative Seebeck coefficients (S), confirming dominant n-type conduction behavior. As the temperature increased, the absolute value of S gradually decreased, which is consistent with the behavior of narrow-bandgap semiconductors.^40^ The power factor (PF), defined in eq 4 as

4 $$\mathrm{PF}=S^{2}\times \sigma$$

where S is the Seebeck coefficient (V/K), and σ is the electrical conductivity (S/m).

As shown in Figure 9(c), Ag_56.6_Se_43.4_, Ag_60_Se_40_, and Ag_63.3_Se_36.7_ reached ∼3.0 mW/mK^2^ due to higher conductivity. Ag_50_Se_50_ and Ag_70_Se_30_, with lower σ, showed lower PF (∼2.3 mW/mK^2^).^41^ Thermal conductivity (κ) and ZT results of the selected best-performing sample (Ag_56.6_Se_43.4_, based on the highest PF) are presented in Figure 9(d). As shown in Figure 9(d), the thermal conductivity decreases monotonically with increasing temperature.

This reduction is attributed to enhanced phonon scattering at elevated temperatures, which is a typical behavior in thermoelectric materials.^42^ In addition, phonon scattering may also be influenced by compositional disorder, defect scattering, and microstructural heterogeneity. It should be noted that grain size effects are not directly verified in this study and therefore cannot be conclusively identified as the dominant factor.^43,44^

Correspondingly, the ZT value increases continuously, reaching a maximum of 1.78 at 390 K. The high ZT value is not only a direct consequence of the high power factor and reduced thermal conductivity, but also originates from the optimized carrier and phonon transport induced by the off-stoichiometric composition. In Ag_56.6_Se_43.4_, the excess Se suppresses Se-vacancy formation, which reduces vacancy-induced carrier scattering and improves carrier mobility while maintaining sufficient carrier concentration.^45,46^ This balance contributes to the simultaneous retention of relatively high electrical conductivity and enhanced Seebeck coefficient. At the same time, compositional disorder, residual Se-related phases, and microstructural heterogeneity provide additional phonon-scattering centers, thereby lowering thermal conductivity.^22^ Furthermore, because ZT is proportional to temperature, the combination of maintained PF and decreasing κ at 390 K further contributes to the maximum ZT value. Therefore, the superior ZT of Ag_56.6_Se_43.4_ results from the synergistic optimization of charge-carrier transport and phonon transport rather than from a single parameter alone. These results confirm that the optimized off-stoichiometric composition effectively enhances thermoelectric performance.

For comparison, previously reported thermoelectric data for stoichiometric Ag_2_Se, measured within a similar temperature range, indicate that the electrical conductivity typically ranges from approximately ∼15 to 22 × 10^4^ S/m.^47^ The corresponding Seebeck coefficient of this work is generally in the range of −80 to 95 μV K^–1^. In contrast, the off-stoichiometric samples in this study exhibit systematically higher absolute Seebeck coefficients, reaching up to −157 μV K^–1^.

Similarly, the power factor of stoichiometric Ag_2_Se is typically reported to be around 12–13 μW cm^–1^ K^–2^ within the investigated temperature range. In comparison, this work samples demonstrate significantly enhanced power factors, reaching up to ∼34 μW cm^–1^ K^–2^, corresponding to an improvement of approximately 2.5–3 times over the stoichiometric baseline.

## 4 Conclusion

This study developed off-stoichiometric Ag–Se alloys and systematically evaluated how compositional imbalance affects their thermoelectric properties. Experimental results revealed that excess Se effectively suppressed the formation of Se vacancies, thereby reducing free carrier concentration and minimizing vacancy-induced electron scattering. As a result, carrier density was regulated, mobility was enhanced, and electrical conductivity improved.

Additionally, grain refinement induced by Se surplus increased phonon scattering at grain boundaries, leading to a reduction in thermal conductivity. Within the composition range of Ag_56.6_Se_43.4_ to Ag_63.3_Se_36.7_, a favorable balance between electrical and thermal transport was achieved.

These findings confirm that composition imbalance engineering is a practical and effective strategy for optimizing Ag_2_Se-based thermoelectric materials. This approach enables simultaneous control over charge transport and thermal resistance by precisely tuning the Ag-to-Se ratio. Overall, this work provides valuable insight into the design of high-performance, low-temperature thermoelectrics and presents a promising pathway for future research in dopant tuning, long-term stability, and device-level integration.
